# Heterotopic ossification of the knee joint in intensive care unit patients: early diagnosis with magnetic resonance imaging

**DOI:** 10.1186/cc5083

**Published:** 2006-10-30

**Authors:** Maria I Argyropoulou, Eleonora Kostandi, Paraskevi Kosta, Anastasia K Zikou, Dimitra Kastani, Efi Galiatsou, Athanassios Kitsakos, George Nakos

**Affiliations:** 1 Department of Radiology, Medical School, University of Ioannina, 45110 Ioannina, Greece; 2 Intensive Care Unit, Department of Internal Medicine, Medical School, University of Ioannina, 45110 Ioannina, Greece

## Abstract

**Introduction:**

Heterotopic ossification (HO) is the formation of bone in soft tissues. The purpose of the present study was to evaluate the magnetic resonance imaging (MRI) findings on clinical suspicion of HO in the knee joint of patients hospitalised in the intensive care unit (ICU).

**Methods:**

This was a case series of 11 patients requiring prolonged ventilation in the ICU who had the following diagnoses: head trauma (nine), necrotising pancreatitis (one), and fat embolism (one). On clinical suspicion of HO, x-rays and MRI of the knee joint were performed. Follow-up x-rays and MRI were also performed.

**Results:**

First x-rays were negative, whereas MRI (20.2 ± 6.6 days after admission) showed joint effusion and in fast spin-echo short time inversion-recovery (STIR) images a 'lacy pattern' of the muscles vastus lateralis and medialis. The innermost part of the vastus medialis exhibited homogeneous high signal. Contrast-enhanced fat-suppressed T1-weighted images also showed a 'lacy pattern.' On follow-up (41.4 ± 6.6 days after admission), STIR and contrast-enhanced T1-weighted images depicted heterogeneous high signal and heterogeneous enhancement, respectively, at the innermost part of the vastus medialis, whereas x-rays revealed a calcified mass in the same position. Overall, positive MRI findings appeared simultaneously with clinical signs (1.4 ± 1.2 days following clinical diagnosis) whereas x-ray diagnosis was evident at 23 ± 4.3 days (*p *= 0.002).

**Conclusion:**

MRI of the knee performed on clinical suspicion shows a distinct imaging pattern confirming the diagnosis of HO earlier than other methods. MRI diagnosis may have implications for early intervention in the development of HO.

## Introduction

Heterotopic ossification (HO) is the formation of bone in soft tissues where it is neither needed nor desired [1]. HO was first described in 1883 by Reidel, and later, in 1918, Dejerne and Ceilliar [2] reported the development of HO among paraplegic patients injured in World War I. Common predisposing conditions for HO are direct muscular trauma, total hip and knee arthroplasty, spinal cord injury, head injury, prolonged sedation, mechanical ventilation, and ankylosing spondylitis [1,3-5]. In critically ill patients, HO has been associated with paralysis and prolonged immobilisation, head and spinal cord injury, acute respiratory distress syndrome (ARDS), burns, and pancreatitis [6]. In the Toronto ARDS Outcomes Study, the prevalence of HO was 5% [7]. Genetically determined forms of HO are fibrodysplasia ossificans progressiva and progressive osseous heteroplasia [1,5]. The pathogenetic mechanism of HO is unknown but probably results from an imbalance between certain forms of bone morphogenetic protein and their antagonists. Overexpression of bone morphogenetic protein in the para-articular soft tissues induces mesenchymal stem cells to differentiate into bone via the enchondral pathway [1,5].

Complications of HO are peripheral nerve entrapment, pressure ulcers, and functional impairment of the joint [5,8]. Surgical resection may be performed to increase joint mobility but should be postponed until the HO matures and becomes less active metabolically [9]. Prevention and early treatment of HO are preferable but these necessitate early detection.

Scintigraphy using technetium-labeled pyrophosphate and ultrasonography (US), which have been used for early detection, are both methods that are sensitive but not specific [10]. Scintigraphy cannot distinguish between early HO and musculoskeletal tumours or infection, whereas US depicts early HO as a non-specific hypoechoic mass [10]. X-rays and computerised tomography scan have also been used, but the earliest findings of HO (a soft tissue mass with peripheral calcification–zone pattern) do not appear until two weeks after clinical suspicion [1,10]. The earliest magnetic resonance imaging (MRI) findings that have been described come from examinations performed 13 ± 18.4 days after clinical suspicion of HO, in the hip joints of patients with spinal cord injury [11]. The purpose of the present study was to evaluate the MRI findings on clinical suspicion of HO in the knee joint of patients hospitalised in the intensive care unit (ICU).

## Materials and methods

This was an observational, prospective case series study that was carried out in the ICU of the University Hospital of Ioannina, Greece, from December 2001 to November 2003. During this period, 670 patients who required prolonged mechanical ventilation (defined as mechanical ventilation for more than seven successive days) were considered eligible for the study. Thirty-one patients developed HO. Eleven consecutive patients with HO of the knee joint who could safely be transported to the radiology department were finally included in the study. From the first day of admission to the ICU, every patient received daily assessment of joint mobility and appropriate passive range-of-motion exercises. Serum levels of alkaline phosphatase (ALP), calcium, and phosphorus were also measured on a daily basis. On the appearance of physical signs (swelling, erythema, or decreased joint motion with or without pain) indicating the development of HO, an x-ray and MRI of the knee joint were performed. X-rays were repeated every 7 days until the appearance of the radiologic signs of HO or earlier if severe deterioration was observed. Patients underwent a second follow-up MRI when x-rays revealed abnormalities compatible with HO. All MRI examinations were performed on the same 1.5-Tesla MR unit (Gyroscan ACS NT; Philips Medical Systems, Best, The Netherlands) using a knee coil, a field of view of 22 cm, an acquisition matrix of 216 × 256, and slice thickness of 4 mm, with an intersection gap of 0.4 mm. Sequences were sagittal and axial fast spin-echo short time inversion-recovery (STIR) with 3,000/80 (repetition time milliseconds/echo time milliseconds) and an inversion time of 165 milliseconds, axial spin-echo plain T1-weighted 500/12 (repetition time milliseconds/echo time milliseconds), and axial, sagittal and coronal contrast-enhanced fat-suppressed (selective partial inversion recovery) T1-weighted spin-echo 650/17 (repetition time milliseconds/echo time milliseconds). X-rays of the knee joint were performed in at least two orthogonal planes. Two senior radiologists (MIA and PK) evaluated independently the x-rays and MRIs for the presence of soft tissue and bone abnormalities. The study was performed under institutional review board approval, and informed consent was obtained for all subjects in the study.

### Statistical analysis

Data analysis was carried out using SPSS Base 14 for Windows (SPSS Inc., Chicago, IL, USA). Differences in the times of appearance of MRI and x-ray findings suggesting HO were evaluated using the Wilcoxon test. A *p *value of less than 0.05 was considered significant. All data are expressed as mean ± standard deviation.

## Results

**Figure 1:**
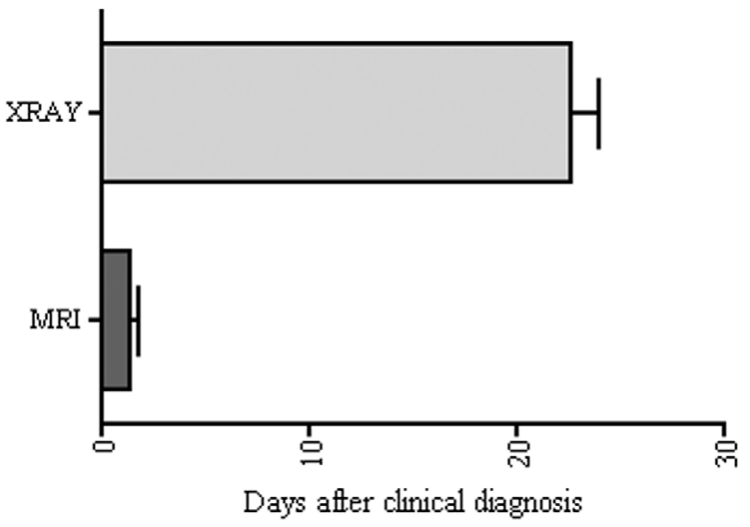
Delay in the emergence of radiological signs after the clinical diagnosis of heterotopic ossification. The difference between magnetic resonance imaging (MRI) and x-ray is statistically significant (*p *= 0.002).

**Figure 2:**
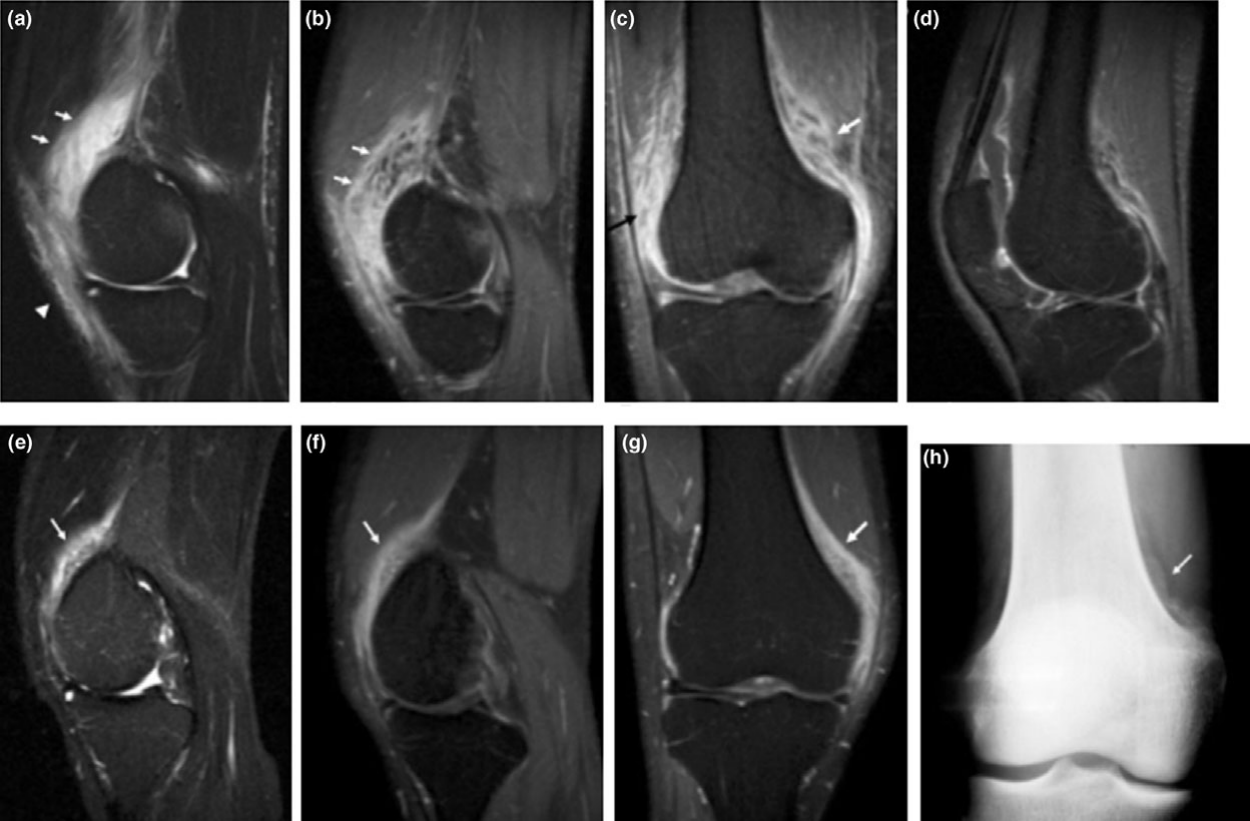
Early and late magnetic resonance imaging (MRI) findings of heterotopic ossification (HO). HO in the right knee of a 35-year-old male patient hospitalised in the intensive care unit for traumatic brain injury. **(a-d) **First MRI performed on clinical suspicion of HO. **(a) **Sagittal fast spin-echo short inversion-recovery MR image (3,000/80; inversion time, 165 milliseconds) shows high signal of the innermost part of the vastus medialis muscle (arrows) and edema of the subcutaneous fat (arrowhead). **(b) **Sagittal contrast-enhanced fat-suppressed T1-weighted spin-echo MR image (650/17) shows a 'lacy pattern' of the innermost part of the vastus medialis muscle (arrows). **(c) **Coronal contrast-enhanced fat-suppressed T1-weighted spin-echo MR image (650/17) shows a 'lacy pattern' of the innermost part of the vastus medialis muscle (white arrow) and vastus lateralis muscle (black arrow). **(d) **Mid-sagittal contrast-enhanced fat-suppressed T1-weighted spin-echo MR image (650/17) shows joint effusion and synovial enhancement. **(e-h) **Follow-up MRI **(e-g) **and x-rays **(h) **performed 3 weeks later. **(e) **Sagittal fast spin-echo short inversion-recovery MR image (3,000/80; inversion time, 165 milliseconds) shows heterogeneous high signal of the innermost part of the vastus medialis muscle (arrow). Sagittal **(f) **and coronal **(g) **contrast-enhanced fat-suppressed T1-weighted spin-echo MR images (650/17) show heterogeneous enhancement of the innermost part of the vastus medialis muscle (arrows). **(h) **Anteroposterior x-ray depicts a calcified mass at the anatomic position of the vastus medialis muscle (arrow).

**Figure 3:**
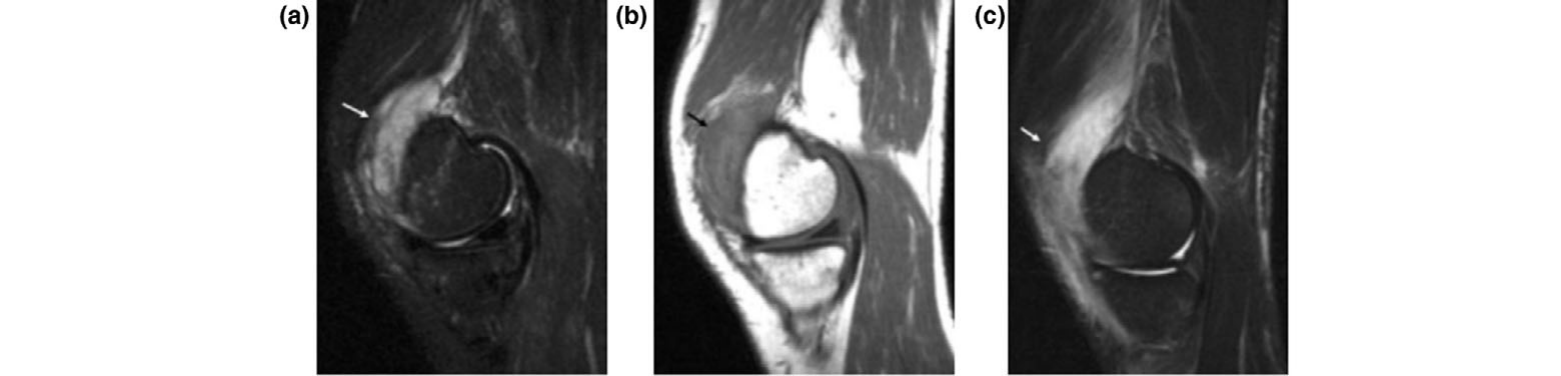
Late magnetic resonance imaging (MRI) findings of heterotopic ossification (HO). HO in the left knee of a 32-year-old male patient hospitalised in the intensive care unit for traumatic brain injury. MRI performed 4 weeks after clinical suspicion of HO. **(a) **Sagittal fast spin-echo short inversion-recovery MR image (3,000/80; inversion time, 165 milliseconds) shows heterogeneous high signal of the innermost part of the vastus medialis muscle (arrow). **(b) **Sagittal T1-weighted spin-echo MR image (650/17) shows heterogeneous high signal of the innermost part of the vastus medialis muscle (arrow). **(c) **Sagittal contrast-enhanced fat-suppressed T1-weighted spin-echo MR image (650/17) shows heterogeneous enhancement of the innermost part of the vastus medialis muscle (arrow).

All of the 11 patients included in the study were male, aged 22 to 70 years (mean 38.2 ± 16.9 years). Admission diagnoses were head injury (nine patients), necrotising pancreatitis (one patient), and fat embolism syndrome (one patient). None of these patients had spinal cord injury and none had received neuromuscular blockade. Early MRI was performed on clinical suspicion of HO (20.2 ± 6.6 days after admission). Table 1 shows the times of appearance of clinical, biochemical, x-ray, and MRI findings suggesting HO. Increase of serum levels of ALP >125 IU/l was the earliest indication of HO (Table 1). On the average, positive MRI findings appeared in all examined patients simultaneously with clinical signs (at 1.4 ± 1.2 days after clinical diagnosis) whereas x-ray diagnosis was evident at 23 ± 4.3 days (*p *= 0.002) (Figure 1). STIR images in all patients demonstrated interstitial edema appearing as septa of high signal intensity in the subcutaneous fat, in the intermuscular fascia, and in the vastus lateralis and vastus medialis muscles. The muscular fibres preserved normal low signal intensity, except for those at the innermost part of the vastus medialis, which exhibited a high signal. Overall, the affected muscles had a 'lacy pattern' and the innermost part of the vastus medialis was homogeneously bright (Figure 2a). Contrast-enhanced fat-suppressed T1-weighted images showed enhancement of the intermuscular fascia and septa, with the muscles again having a 'lacy pattern,' even at the innermost part of the vastus medialis (Figure 2b,c). Intra-articular fluid was also present, appearing with a high signal in the STIR images. Synovial enhancement was observed on contrast-enhanced T1-weighted images (Figure 2d). X-rays of the knee joint synchronous to the first MRI did not reveal any abnormality. Follow-up MRI performed 41.4 ± 8.2 days after admission revealed restriction of the lesion to the innermost part of the vastus medialis, which showed a heterogeneous high signal in STIR images (higher than that of normal muscle), in T1-weighted images, and heterogeneous enhancement in contrast-enhanced fat-suppressed T1-weighted images (Figures 2e–g and 3a–c). At the same time as the follow-up MRI, x-rays depicted a calcified mass located close to the bone at the anatomic position of the innermost part of the vastus medialis (Figure 2h) (Table 1). In all cases, there was agreement (consensus) in the interpretation of x-rays and MRIs.

## Discussion

The main finding in this study was the early MRI findings suggesting HO. MRI was always performed soon after the first clinical suspicion of HO. X-rays performed at the same time revealed no abnormality. To the best knowledge of the authors, the 'lacy pattern' in the affected muscles on MRI is the earliest radiological finding associated with HO and it is described here for the first time.

There are only a few case reports describing MRI findings of HO in the knee joint [12,13]. In those cases, MRI was performed six weeks to three months after the onset of clinical symptoms and the findings were similar to those observed at follow-up MRI in the patients in the present study. Systematic studies have been conducted only for evaluation of HO in the hip joint [11,14]. The MRI pattern of HO described in other studies is different from that observed in the cases in the present study. This difference is probably because the first MRI in the present study was performed early, on clinical suspicion, whereas the MRIs in previous studies were performed later in the course of the disease. Ledermann *et al*. [14] evaluated a series of bedridden paralysed patients by MRI of the pelvis performed 10.6 ± 8.93 years after the onset of paralysis. According to their study, (a) immature HO appeared in STIR images with hypersignal and in T1-weighted images with isosignal to normal muscle, enhanced after contrast administration, and (b) mature HO appeared in STIR images with hyposignal to normal muscle and in plain T1-weighted images with isosignal to the fatty marrow and did not enhance after contrast infusion [14]. Wick *et al*. [11] described the MRI findings of HO of the hip joint based on examinations performed 13 ± 18.4 days after clinical suspicion, in a series of paralysed patients with spinal cord injury. According to these authors, HO in muscles exhibits hypersignal in T2-weighted images and hyposignal in T1-weighted images and does not enhance after contrast administration [11]. In the present study, MRI performed on clinical suspicion of HO depicted a 'lacy pattern' in the vastus lateralis and vastus medialis muscles in both contrast-enhanced T1-weighted and STIR images (except for the innermost part of the vastus medialis, which exhibited a homogeneous high signal on STIR images). This was the only part of the vastus medialis involved in the development of HO.

In previously reported cases of HO in the knee joint, precipitated by either trauma or neurogenic causes, the lesion was located at the anatomic position of the vastus medialis muscle [3,12,13,15]. In agreement with these previous studies, in the present series of bedridden ICU patients, HO affected the vastus medialis muscle and particularly its innermost portion. Several studies have demonstrated that gravitational unloading due to oxidative stress causes muscle atrophy [16-20]. Slow-twitch muscle fibres develop greater atrophy than fast-twitch muscle fibres [16-20]. This is probably because slow-twitch muscle fibres, which are aerobic fibres and are normally provided with energy from the Krebs cycle, are at disadvantage under anaerobic conditions [21]. The vastus medialis, in its deep portion, close to the bone, contains slow-twitch fibres, which after exposure to weightlessness show increased lactate dehydrogenase activity and develop the most severe atrophy [18]. Anaerobic conditions with low local oxygen concentration also promote bone cell proliferation and therefore might play a role in the development of HO in slow-twitch muscle fibres [22].

Central nervous system or local traumatic injuries are well-recognised predisposing conditions for the development of HO [1,3-5]. This study demonstrated the development of HO in previously normal knee joints, not only in patients with brain injury but also in two patients with no overt predisposing neurogenic factor. These two patients were under long-term sedation and mechanical ventilation. Previous studies have demonstrated that sedation and mechanical ventilation may play a role in the development of HO by inducing a pathogenic condition similar to neurogenic HO and by causing changes in the local tissue PO_2 _(partial pressure of oxygen) and pH [3,4,22]. Apart from local disturbances in tissue oxygenation and pH, other factors such as bone morphogenetic protein have also been implicated in the pathogenesis of HO [23,24]. According to the current concept, bone morphogenetic protein, which is released from normal bone under conditions that often accompany trauma and immobilisation, induces the development of bone from mesenchymal cells found in the intermuscular septa [24].

Early MRI findings of HO should be differentiated from the following: (a) Muscle necrosis is usually a focal process that has been associated with diabetes and alcoholism. Patients present with acute painful muscle swelling, and the affected muscle shows a peripheral enhancement that was not observed in the present cases of early HO [25]. (b) Pyomyositis is a primary bacterial infection affecting skeletal muscle, occurring more frequently in diabetic and immunocompromised patients [26]. In the early purulent stage of pyomyositis, a 'feather-like' pattern has been described that is in contrast to the homogeneous high signal observed in STIR images at the innermost part of the vastus lateralis muscle in early HO in the present series. (c) Soft tissue tumours appear as masses within a single muscle or multiple muscle groups and tend to infiltrate and disrupt fascial planes in contrast to HO, in which intermuscular fascia are preserved [25].

Late MRI findings should be differentiated from the following: (a) Pellegrini-Stieda disease is a post-traumatic ossification proximal to the medial femoral condyle. Ossification in Pellegrini-Stieda disease follows the course of the medial collateral ligament and therefore extends further down than that in HO [27]. (b) Parosteal osteosarcoma is a low-grade, bone-forming metaphyseal tumour characterised by thickening of the cortex and a mineralised soft tissue component. Findings that are helpful in distinguishing parosteal osteosarcoma from HO are the lack of cortical thickening and the presence of a cleavage plane between the bone and the calcified lesion in the latter [28].

Early detection of HO in critically ill patients is a clinical challenge. The symptoms and signs are non-specific, and clinical diagnosis is often delayed because of the sedation and immobilisation of the patients. The laboratory findings, such as an increase in serum ALP, are also non-specific. HO can be a cause of fever in ICU patients and may mimic septic arthritis. With every new episode of fever, ICU patients are often exposed to a variety of diagnostic procedures and inappropriate antimicrobial treatment. 'In advance' diagnosis of HO with MRI could potentially reduce these unnecessary risks and costs.

Survivors of critical illness, and especially of ARDS, have persistent functional disability and exercise limitation due to neuromuscular weakness, nerve entrapment syndromes, and large-joint immobility due to HO [7]. Although the treatment of HO remains a controversial issue, there is probably a place for prophylactic treatment involving non-steroidal anti-inflammatory drugs or single-dose irradiation [29]. Both of these treatment options, which have been studied in patients with total hip replacement, possibly act by suppressing early inflammatory changes. To the best of our knowledge, there is no evidence concerning the effect of these treatment options on HO related to critical illness. Nevertheless, any intervention to improve functional outcomes should be based on an effective screening method. In this context, early diagnosis with MRI might present an important advantage, which is to facilitate effective prevention of HO.

In the present study, we described the MRI findings of HO in the knee joint in a series of 11 patients. This was a small-scale, albeit prospective, study that disclosed the capability of MRI to detect early HO changes. From the clinical point of view, the risks of transporting critically ill patients and the cost of the method could pose limitations in the wider application of this diagnostic modality. Further studies are needed to examine the potential therapeutic and quality of life benefit associated with an early diagnosis of HO.

## Conclusion

The early MRI findings of HO in the knee joint are interstitial edema of the subcutaneous fat, thickening of the intermuscular septa, joint effusion, and a 'lacy pattern' of the vastus lateralis and vastus medialis muscles, except for the innermost part of the vastus medialis, which exhibits a homogeneous high signal in STIR images. HO develops at the innermost part of the vastus medialis, which is composed of aerobic slow-twitch fibres that are at a disadvantage under anaerobic conditions of weightlessness. These MRI findings confirm the clinical suspicion of HO, whereas joint x-ray is negative. MRI diagnosis may have implications for early intervention in the development of HO.

## Key messages

• MRI of the knee performed on clinical suspicion of HO shows a distinct imaging pattern consisting of a 'lacy pattern' of the muscles vastus lateralis and medialis.

• These MRI findings confirm the clinical suspicion of HO whereas x-ray is negative.

• MRI diagnosis may have implications for early intervention in the development of HO.

## Abbreviations

ALP = alkaline phosphatase; ARDS = acute respiratory distress syndrome; HO = heterotopic ossification; ICU = intensive care unit; MRI = magnetic resonance imaging; STIR = short inversion-recovery; US = ultrasonography.

## Competing interests

The authors declare that they have no competing interests.

## Authors' contributions

MIA served as guarantor of integrity of the entire study and conducted literature research, clinical studies, statistical analysis, and manuscript editing. EK and PK carried out literature research, clinical studies, and manuscript editing. AKZ, DK, and AK conducted literature research and clinical studies. EG performed clinical studies, statistical analysis, and manuscript editing. GN served as guarantor of integrity of the entire study and conducted literature research and manuscript editing. All authors read and approved the final manuscript.

## Figures and Tables

**Table 1:** Times of appearance of clinical, biochemical, MRI, and x-ray findings suggesting HO

Findings indicating HO	Clinical	ALP	MRI	X-rays
Time in days mean ± standard deviation	18.7 ± 6.7	13.6 ± 4.7	20.2 ± 6.6	41.4 ± 8.2

Heterotopic ossification (HO) in the knee joint of 11 intensive care unit patients. Increase of serum levels of alkaline phosphatase (ALP) was the earliest indication of HO. MRI, magnetic resonance imaging.
